# Emerging Trends in Neuromodulation for Treatment of Drug-Resistant Epilepsy

**DOI:** 10.3389/fpain.2022.839463

**Published:** 2022-03-21

**Authors:** Mohamed Abouelleil, Nachiket Deshpande, Rushna Ali

**Affiliations:** ^1^Division of Neurological Surgery, Spectrum Health, Grand Rapids, MI, United States; ^2^College of Human Medicine, Michigan State University, East Lansing, MI, United States

**Keywords:** deep brain stimulation, responsive neurostimulation, vagus nerve stimulation, epilepsy, anterior nucleus of thalamus, pulvinar, centromedian nucleus of thalamus, Trigeminal Nerve Stimulation (TNS)

## Abstract

Epilepsy is a neurological disorder that affects more than 70 million people globally. A considerable proportion of epilepsy is resistant to anti-epileptic drugs (AED). For patients with drug-resistant epilepsy (DRE), who are not eligible for resective or ablative surgery, neuromodulation has been a palliative option. Since the approval of vagus nerve stimulation (VNS) in 1997, expansion to include other modalities, such as deep brain stimulation (DBS) and responsive neurostimulation (RNS), has led to improved seizure control in this population. In this article, we discuss the current updates and emerging trends on neuromodulation for epilepsy.

## Introduction

Neuromodulation has come to the forefront as a novel and effective treatment modality for neurological diseases. It involves directly stimulating or impeding neuronal action potential conduction (1). This can be accomplished through various mechanisms, including chemical, mechanical, thermal, optogenetic, magnetic, and electrical manipulation, with electrical stimulation being the most widely used (1).

Notably, neuro-modulatory interventions have emerged as a pivotal alternative in the management of medically refractory epilepsy for patients who are not candidates for resection or ablation (2). The prevalence of epilepsy in the general population is estimated to be ~0.5–1%, with 30% of these patients being resistant to medical therapy (3, 4). In 2010, the International League Against Epilepsy defined drug resistant epilepsy as failure to attain seizure control using two adequate trials of appropriately selected and utilized anti-epileptic medication regimens (5). Epilepsy can be classified into the following subtypes: generalized, focal, combined generalized and focal, and unknown epilepsy (6, 7). The etiology of refractory epilepsy is complex. The thalamus is a key subcortical structure implicated in the epilepsy network, making various nuclei desirable targets for neuromodulatory techniques, such as deep brain stimulation (DBS) and responsive neurostimulation (RNS).

## Candidates for Neuromodulation

After 1 year of unsuccessful seizure control with the use of two or more anti-epileptic medications at adequate dose, patients should be referred to comprehensive epilepsy centers. Patients with refractory epilepsy that are good candidates for neuromodulation include those who have seizure foci involving the eloquent cortex, decline traditional surgical resection, have multifocal or generalized epilepsy, or have continued seizures despite resection/ablation. It has been reported that 30–40% of patients with temporal lobe epilepsy do not have adequate seizure control after resection (8), and one systematic review found only 27–46% seizure freedom with extratemporal resections (9). These shortcomings with resective surgery highlight the key role of neuromodulation in management of drug resistant epilepsy.

## Neuromodulation Techniques

### VNS

Stimulation of the cervical vagus nerve with high frequency and low voltage has been shown to induce synchronization on EEG, with increasing voltage leading to desynchronization (10). Vagal nerve stimulation (VNS) has also been shown to significantly increase the inhibition of circuits in the motor cortex by increasing the activity of GABA_A_ receptors, supporting the notion that neurotransmitter modulation is an underlying mechanism in seizure termination. The locus coeruleus (LC) and dorsal raphe nucleus (DRN) are two destinations for the nucleus tractus solitarius (NTS), a location where a considerable number of vagal afferents terminate. Consequently, the LC and DRN have been implicated in the mechanism of VNS. VNS has been shown to increase noradrenergic and serotonergic activity, from the LC and DRN, respectively, and reduce seizures in rat models (11).

In 1997, the Food and Drug Administration (FDA) approved the use of implantable vagus nerve stimulators for the treatment of drug resistant focal onset impaired awareness seizures in patients older than 4 years of age after an open label study demonstrated seizure reduction of >50% in 36.8% of patients 1 year after implantation, 43.2% of patients at 2 years, and 42.7% at 3 years (12). Reported adverse events were hoarseness (28%) and paresthesia (12%) at 1 year, hoarseness (19.8%) and headache (4.5%) at 2 years, and shortness of breath (3.2%) at 3 years. In a recent meta-analysis, 74 studies and 3,321 patients with intractable epilepsy were identified (13). VNS reduced the frequency of seizures by an average of 45%, 36% at 3–12 months and 51% after 1 year. The study also demonstrated the extension of VNS benefits to patients with generalized epilepsy, who experienced a reduction of 57.5%. Furthermore, patients with tuberous sclerosis and post-traumatic epilepsy benefited the most, with seizure reduction of 68 and 79%, respectively.

Novel advancements in VNS technology have allowed for the utilization of a closed loop stimulation approach in VNS using a heart rate-based seizure detection algorithm (14). Further, improvements in VNS programming, such as the ability to personalize therapy according to time of day, have enhanced the capability of VNS to be tailored to each patient's needs. Recent studies have compared efficacy between open and closed loop VNS (15, 16). One retrospective study found that closed loop VNS was associated with a greater reduction in seizure frequency compared to open loop VNS at 9 months following implantation (15); however, this difference was no longer present at 24 months after implantation. Another study comparing open and closed loop VNS in pediatric patients reported similar efficacy between the two in reducing seizure frequency (16). These findings suggest that open and closed loop VNS are both viable options for refractory epilepsy.

### TNS

Trigeminal Nerve Stimulation (TNS) is a non-invasive, transcutaneous stimulation modality for medication-resistant epilepsy. Mechanistically, TNS exerts its effects through modulation of the trigeminal nucleus and its projections. Afferents from the trigeminal nerve synapse onto the NTS and LC (17), which are two brain regions that have been previously implicated in playing a role in seizure reduction and are targeted using VNS as well (11).

In 1976, pioneering work by Maksimow illustrated that application of pressure on infraorbital branches of the trigeminal nerve can inhibit generalized tonic clonic seizures if done prior to the beginning of convulsions (18). Following this, several studies supported the notion that TNS can reduce seizure frequency (19–21). The strongest evidence for safety and efficacy of TNS in refractory epilepsy stems from a double blind randomized controlled trial of 50 patients (22). At the conclusion of the 18-week blinded period, 30.2% of the stimulation arm and 21.1% of the control arm had a reduction in seizure frequency of >50% (responder rate). Additionally, the stimulation group had an increase in responder rate over the 18 weeks that was not seen in the control group. Side effects of TNS during the study period included skin irritation (14%), headache (4%), and anxiety (4%) (22). In a separate study investigating TNS safety, no short- or long-term cardiovascular side effects were seen with TNS (23).

In contrast to other neuromodulatory techniques, TNS does not require implantation of hardware. This is a key advantage of this modality and is ideal for poor surgical candidates. Furthermore, TNS has been linked with improvements in mood (22, 24) and could be beneficial for patients with co-morbid depression.

### Closed Loop Stimulation

Responsive neurostimulation (RNS) is the initiation of stimulation in response to the detection of an epileptic event electrographically and is known as a closed-loop neuromodulation system. Of great significance to RNS, is the work of Psatta (25). His work demonstrated that responsive stimulation was more effective than continuous, open-loop stimulation in terminating epileptic activity in cat models. He also highlighted the temporal importance of stimulation; stimulation was more effective when applied at shorter intervals after detection of epileptic patterns (25). The later works of Motamedi et al. introduced the significance of spatial stimulation in seizure suppression. They found that stimulation of seizure onset zones was most effective in terminating after-discharges, supporting the notion that direct stimulation of epileptic foci enhances effectiveness in terminating seizure propagation (26).

RNS systems were approved by the FDA in 2013 for the treatment of drug resistant partial onset epilepsy in patients above the age of 18 years who are refractory to ≥2 trials of antiepileptic medications, have ≤2 epileptogenic foci, and experience significant impairment due to frequent seizure activity.

Closed-loop stimulation leads to the termination of synchrony in seizure onset zones to prevent propagation of the ictal stage. Electrical stimulation of these foci during seizure onset leads to alterations in cell membrane currents and hyperpolarization rather than desynchronization (27, 28). Axonal conduction, depression, and blockade are also hypothesized to be underlying mechanisms (29). Finally, chronic stimulation has been shown to alter gene expression, leading to cortical reorganization and synaptic plasticity that could further enhance the anti-epileptic effects (30).

A 9-year prospective study evaluated the efficacy and safety of RNS in patients with intractable focal onset epilepsy. Two hundred and thirty patients were recruited and 162 were able to complete the 9-year follow-up period (31). The percentage of seizure reduction was recorded at intervals of 6 months and median seizure reduction was 75%. Furthermore, 21% of patients achieved seizure freedom and greater than one third of patients achieved >90% reduction. Overall, seizure reduction percentages improved over time following implantation. Patients also reported improvement in their quality of life and perception of cognition, compared to temporal lobe resection and selective amygdalohippocampectomy.

Complications were no more significant than other treatment methods for epilepsy, including VNS, DBS, and surgical resection. Infection rate was 4.1% per procedure and 12.1% for the 1,895 patient implantation years. All infections were in the soft tissue and involved skin flora. 2.7% of patients experienced non-seizure related hemorrhage without neurological deficits. There were no exacerbations in depression, suicidality, or memory impairment (31).

### Open Loop Stimulation

In the early 1900's, Clarke and Horsley were the first to develop a stereotaxic apparatus that could facilitate targeted exploration of the deep ganglia and tracts (32). Although electrical stimulation was used in such operations, its main objective was to confirm the target areas prior to lesioning. Exploration and utilization of chronic DBS as a therapeutic rather than diagnostic intervention quickly ensued (33). Utilization of DBS in a wide range of conditions, such as essential tremor, Parkinson's disease, and pain, built the safety profile of this intervention, leading to a rapid decline in lesioning interventions. The success of DBS in movement disorders drew attention to its use in refractory epilepsy.

In 2018, the FDA approved the use of DBS for patients with refractory partial-onset seizures, with and without generalization, who are 18 years of age or older. Contraindications for DBS are minimal. DBS is contraindicated in patients who are incapable of operating the neurostimulator, have significant psychiatric contraindications, or are medically frail and unable to undergo surgical implantation (34).

The anti-epileptic mechanisms of DBS are largely unknown and complex. The proposed underlying mechanisms include inhibition, likely due to blockade of depolarization and voltage gated currents, or activation of GABAergic neurons (35–37). Shifting the focus from the effects of DBS on neurons, some studies highlighted the role of astrocyte activation in DBS by inducing local neuronal modulation (38). Electrical currents by DBS have been shown to induce electrotaxis- the migration of progenitor cells due to electricity- which can serve to promote neurogenesis and neuroplasticity that could alter neuronal pathways (39).

Most of the data regarding the safety and efficacy of DBS in epilepsy patients stems from the results of SANTE trial (40). The SANTE trial was a randomized, double blinded clinical trial investigating anterior nucleus of thalamus stimulation for epilepsy and consisted of a 3-month blinded study period. DBS implantation itself resulted in seizure reduction in both groups. Initial median seizure reduction was 33.9% in the active stimulation arm and 25.3% in the control arm in the first month following DBS implantation. However, this difference between groups expanded further during the final month of the blinded phase; seizure reduction diminished in the control arm to 14.5%, while the treatment arm exhibited a 40.4% reduction compared to baseline. Stratification by lobe of onset during a *post-hoc* analysis demonstrated a significant reduction rate for the temporal lobe (43.9% treatment vs. 29% control) that was not observed for other lobes (41). Additionally, patients with complex partial seizures benefited more than patients with simple partial seizures. A long-term follow-up study evaluating the safety and efficacy of DBS in the SANTE cohort demonstrated continued improvement of efficacy overtime (42); Specifically, a 41% and 69% median seizure reduction from baseline was noted after DBS at one and five years, respectively, in the SANTE cohort. At 5 years following surgery, differences in seizure reduction were present when stratifying by lobe of onset. Those with temporal lobe epilepsy experienced the greatest median seizure reduction (76%), while those with frontal lobe epilepsy experienced the least seizure reduction (59%).

Adverse effects over a follow-up period of 5 years included paresthesia and pain at the site of implants (20.9%), implant site infections (12.7%), and improper lead positioning (8.2%). A possible stimulation related adverse effect was depression (32.7%); however, 66% of patients had pre-operative depression (42).

### Thalamic Stimulation Targets for Medically Refractory Epilepsy

The thalamus is a sensory relay center with widespread synaptic connectivity to many cortical regions. The role of this key subcortical structure in the propagation of abnormal epileptiform activity has long been described (43). Direct electrical stimulation of the thalamus allows for modulation of neural circuitry and ultimately interferes with seizure propagation. The specific anatomical and physiological profiles of various thalamic nuclei, detailed in the subsections below, make them attractive targets for DBS and RNS in epilepsy patients.

While DBS of the anterior and centromedian thalamic nuclei for epilepsy have been studied extensively, a novel promising target is the median pulvinar thalamic nucleus. Additionally, most studies evaluating the effectiveness and safety of RNS are limited to cortical stimulation, with less being known about subcortical targets.

#### Anterior Thalamic Nucleus

One of the well-known targets of thalamic stimulation is the anterior nucleus of the thalamus (ANT). The specific therapeutic target within this nucleus is the ventral ANT, which is located 4–6 mm lateral to mid commissural point, 1–2 mm anterior to the mid commissural point, and 10–12 mm superior to the Anterior Commissure-Posterior Commissure (AC-PC) plane. The ANT is a key component of the Papez Circuit, whose role in seizure propagation has been extensively explored. In this circuit, the ANT receives afferents mainly from the mamillary bodies, the hippocampus, and mesial temporal region. The ANT projects diffusely in the cerebral cortex, including the cingulate gyrus and the lateral temporal cortex (44). Mirski and Ferrendelli compiled evidence from three main studies regarding the involvement of Papez Circuit in seizures. Severing the mammillothalamic tract in guinea pigs prevented the induction of seizures by pentylenetetrazole (PTZ) (45). Mirski and Fisher were also able to increase seizure threshold in rats by stimulating the mamillary bodies (46). High frequency stimulation of the ANT in rats provided significant protection against PTZ induced seizures (44).

Prior to the completion of the SANTE clinical trial, many smaller scale studies evaluated the efficacy of ANT DBS. In 1987, Upton et al. were the first to demonstrate the therapeutic benefit of ANT DBS in reducing seizure activity, with four of the six study patients experiencing clinical control of their epilepsy following stimulation (47). Following these promising results, several similar size studies reaffirmed the role of ANT stimulation in reducing seizure frequency (48–52). The long-term effect of ANT DBS has also been reported, with the SANTE cohort demonstrating continued safety and efficacy during the 5 years of follow up (42). Importantly, quality of life in this cohort was also reported to be significantly improved 5 years after implantation. Recently, a study evaluating long term ANT DBS effectiveness was completed by Kim et al. who reported a 60–80% reduction in seizure frequency in the 11 years following DBS implantation (53). Given the substantial evidence supporting ANT DBS, it is a highly desirable and routinely utilized target for focal epilepsy.

Compared to DBS, less data is available on the results of ANT RNS for epilepsy. Herlopian et al. reported a case of a 34-year-old male with generalized epilepsy who suffered from tonic, atonic, myoclonic, and absence seizures that frequently generalized since the age of 3 (54). The patient underwent corpus callosotomy and then VNS, which was later removed due to inefficacy. RNS in the bilateral posterior frontal cortex as well as the bilateral ANT reduced seizure frequency by 90–95%, from 15–20/day to 2–3/day. Responsive stimulation of unilateral ANT for multifocal epilepsy has also been described (55). In this case series of 3 patients who underwent a combination of bilateral and unilateral ANT RNS, 2 patients experienced >50% seizure reduction while the other patient had 50% reduction at 33 months follow-up. There were no adverse effects on behavior, mood, or memory. These encouraging results from subcortical RNS, combined with its safety and efficacy profiles, suggest that RNS may serve as an alternative to deep brain stimulation (DBS), which is an open-loop system.

#### Centromedian Nucleus

An increasingly popular thalamic target for neuromodulation is the centromedian nucleus (CM). Within the CM, the therapeutic target is the dorsolateral CM, which is located 8–10 mm lateral to the mid commissural point, 1 mm anterior to the posterior commissure, and at the AC-PC line. The CM receives input from the motor cortex and basal ganglia and projects to the motor cortex and striatum. It is involved in cognition, sensorimotor coordination, and arousal (56, 57). The CM is a desirable target for neuromodulation due to its connectivity with the anterior cingulate gyrus, which is part of the Papez Circuit and implicated in seizure propagation (56, 57).

Velasco et al. were the first to use DBS of the bilateral CM in drug-resistant epilepsy and noted a significant reduction in seizures after stimulation (58). This was followed by the work of Fisher et al. who conducted a double blinded, placebo controlled cross-over study of seven patients with intractable epilepsy (59). Patients underwent bilateral DBS of the CM and stimulation was activated in blocks of 3 months, with a 3 month off period. Tonic-clonic seizure reduction of 30% was observed when stimulation was on, compared to 8% reduction when stimulation was off. Subsequently, Velasco et al. conducted an open label clinical trial further evaluating the effectiveness of CM stimulation (60). Of the 13 patients who received this intervention, patients with generalized tonic-clonic seizures and Lennox-Gastaut syndrome had a significant decrease in seizure frequency and benefited the most (57.3 and 81.6% reduction, respectively). Unfortunately, these benefits did not extend to patients with partial onset or temporal seizures. In a study of 11 patients, five with frontal lobe seizures and six with generalized epilepsy, only one patient in the frontal lobe group had clinical improvement. In contrast, 100% of the generalized epilepsy patients exhibited reduction in seizure frequency (61). Although further studies and results are warranted, preliminary data on CM stimulation support its efficacy in treating generalized rather than focal or temporal epilepsy. Recently, Son et al. chronically stimulated CM in Lennox-Gastaut Syndrome and multilobar epilepsy patients (62). They reported a mean 68% seizure reduction and of the 14 study patients, 11 experienced >50% reduction in frequency (4/4 in Lennox-Gastaut and 7/10 in multilobar epilepsy).

Effective use of RNS for CM stimulation has also been described. Welch et al. demonstrated effectiveness of bilateral CM RNS in treatment of primary generalized epilepsy and childhood absence seizure in a 16-year-old male patient (63). The patient achieved a complete resolution of detectable absence seizures and a 75% reduction in convulsive seizures at 6 months follow-up. The authors hypothesized that CM stimulation prevented the low frequency thalamocortical epileptic propagation. CM RNS has also been shown to be effective in treatment of Jeavons Syndrome (64) and epilepsy of regional neocortical onset (65).

#### Pulvinar

Although the involvement of the lateral pulvinar in visual processing is well-studied, less is known about the medial pulvinar (PM), which is the therapeutic target for epilepsy. Using susceptibility weighted imaging (SWI) or T1 magnetic resonance imaging (MRI), the PM can be located at 10–12 mm lateral to mid commissural point, 3–5 mm posterior to the posterior commissure, and 0-3 mm superior to AC-PC plane. It is well-established now that the pulvinar contains a multitude of nuclei with functions that extend beyond visual processing. Many studies have explored the broad reciprocal connections between the medial pulvinar and the neocortex (66). The PM is also involved in working memory, attention, and executive function. Abnormal PM connectivity and function has been associated with attention deficit hyperactivity disorder (ADHD) as well as schizophrenia (66). Based on the extensive connectivity of the PM to the cerebral cortex, Rosenberg et al. studied its involvement in the propagation of temporal lobe epilepsy (67). They observed that the PM exhibited ictal activity that corresponded with the onset of temporal lobe seizures, suggesting its role in their propagation. Such findings led to subsequent studies that explored PM stimulation in treating refractory epilepsy.

Although data on pulvinar DBS for epilepsy is scarce, its reciprocal functional connectivity to the cerebral cortex was examined in seven epileptic patients (68). Cortical evoked potential response to medial pulvinar stimulation was 80% in the temporal neocortex, temporo-parietal junction, the insula, and the frontoparietal operculum. The PM response to cortical stimulation was most extensive in mesial temporal region (80%), temporal cortex (76%), and the temporo-parietal region (67%). In comparison, the frontoparietal operculum and insula induced a 14% response. These findings support the extensive, asymmetrical, reciprocal connectivity of the PM to the cerebral cortex (68). Furthermore, retrospective imaging studies of patients with focal onset status epilepticus demonstrated involvement of the PM (69). Diffusion weighted imaging (DWI) revealed thalamic restriction in 20 of the 33 temporal status epilepticus cases. Of these 20 cases, 18 involved the PM. The PM was significantly less involved in parietal and frontal onset status epilepticus. Filipescu et al. studied the effect of PM stimulation on temporal lobe epilepsy in eight patients undergoing stereoelectroencephalography (SEEG) (70). Diagnostic stimulation involving the hippocampus was accompanied by ipsilateral stimulation of the PM. Seventeen seizures were induced and five out of the eight patients experienced less severe seizures when PM stimulation was on, especially with regard to alteration of consciousness. In conjunction with these results, RNS of the pulvinar was successful in treating posterior quadrant epilepsy (71). Of note, an open label clinical trial (Pulvinar Stimulation in Epilepsy: a Pilot study) is currently underway evaluating the effectiveness of PM stimulation on seizure reduction, with recruitment beginning in early 2021.

Currently, one study has evaluated outcomes of PM RNS in epilepsy. Burdette et al. reported successful treatment of three patients with drug-resistant regional onset epilepsy using responsive neurostimulation of the pulvinar nucleus (71). Seizure onset regions were the occipital and posterior temporo-parietal regions. At 1 year follow-up, all patients were responders (achieved 50% or greater reduction in seizures) and two patients achieved >90% reduction. These findings support the involvement of pulvinar in the propagation of posterior quadrant epilepsy.

## Combined Stimulation

The RNS and VNS safety and efficacy profiles are not dissimilar. One retrospective study compared the efficacy and safety of RNS vs. VNS and found that no significant differences exist (72). Thirty patients with refractory epilepsy underwent either VNS or RNS at a single institution. Seizure reduction rates were comparable, 66% (VNS) and 58% (RNS). Similarly, minor complications occurred: 15% (VNS) and 18% (RNS). Neither group had significant morbidity or mortality. Another single institution retrospective study reported similar outcomes between VNS and RNS in 23 patients with temporal lobe epilepsy (73). Less is known about the efficacy of combining both interventions. In fact, initial RNS studies for FDA approval excluded patients who had a VNS system in place. Preliminary studies demonstrated a synergistic effect of combined VNS and RNS. Two patients received RNS in addition to prior VNS, with one patient having history of bilateral mesial temporal epilepsy and another with bilateral hippocampal sclerosis. The combined effect of both systems was tested by deactivating the VNS system, which led to increased clinical and electrographic seizures (74). These findings suggested a synergistic relationship when the two systems are combined.

Combined open and closed loop deep brain stimulation has been studied in rodent models. One study evaluated seizure frequency reduction in rats that received closed loop stimulation followed by open loop stimulation compared with rats that received no stimulation (75). It found a 90 and 17% decrease in seizure frequency with closed and open stimulation, respectively, when compared to rodents that received no stimulation. One case study has reported on a patient that had an RNS system previously implanted and later also received ANT DBS (76). This dual system approach allowed investigators to highlight the influence of ANT stimulation on hippocampal activity by utilizing RNS system electrocorticography. They found that ANT DBS suppressed hippocampal epileptiform activity and modulated connectivity between the hippocampus and neocortex. This combined RNS-DBS approach could pave the way for future neuromodulatory systems that are able to incorporate the two systems into one new device.

Although several studies have examined stimulation of a single thalamic nucleus (Table 1) (81), the literature examining the impact of concurrent stimulation of multiple thalamic nuclei on seizure frequency is sparse. Hu et al. reported a mean 63% reduction in seizure frequency after bilateral ANT and CM DBS in four patients that were refractory to VNS and/or resective surgery (82). Recently, one study retrospectively compared the effects of CM DBS with and without simultaneous ANT stimulation (83). They noted no significant difference in reduction of seizure frequency between the CM + ANT group (60% reduction) and CM only group (56% reduction). While both groups demonstrated similar safety and efficacy, future larger scale studies must follow to draw substantial conclusions on the effects of stimulation of more than one thalamic nucleus on seizure control.

**Table 1:** The effect of open and closed loop stimulation on seizure frequency.

	**References**	**#Subjects**	**Seizure type**	**Seizure reduction**
**Open loop stimulation**				
ANT	Upton et al. (47)	6	CP	Seizure control in 4/6
	Hodaie et al. (51)	5	GTC, CP, DA, SGTC, AA, partial motor	Mean 54% reduction in first year post-DBS
	Kerrigan et al. (49)	5	CP, SGTC, SP	Mean ~50% “serious seizure” reduction
	Andrade et al. (52)	6	GTC, CP, DA, SGTC, AA, partial motor	≥50% reduction in 5/6 in years 2–7 post-DBS
	Lim et al. (77)	4	GE, P, STGC	Mean 49% reduction
	Osorio et al. (48)	4	CP, SGTC, DA, SP, Bitemporal Mesial	Mean 75.6% reduction
	Fisher et al. (40)	54	CP, SGTC	Median 56% reduction at 2 years post-DBS
	Lee et al. (50)	15	SP, CP, GTC	Mean 70.4% reduction
	Salanova et al. (42)	83	CP, SGTC	Median 69% reduction at 5 years post-DBS
	Järvenpää et al. (78)	16	Multifocal, T, F, PA	≥50% reduction in 12/16
	Järvenpää et al. (79)	27	SP, CP, SGTC	Mean 65% reduction at 5 years post-DBS in CP
CM	Velasco et al. (58)	5	CP, GTC, DA, Myoclonic	Significant reduction in seizures with stimulation
	Fischer et al. (59)	7	GTC	Mean 30% Reduction
	Velasco et al. (60)	13	GTC, AA, DA, CP, SGTC, LGS	57.3% reduction (SGTC) & 81.6% reduction (LGS)
	Valentín et al. (61)	11	GE, F	≥50% reduction in 1/5 (GE) & 5/5 (F)
	Son et al. (62)	14	LGS, SP, CP, GTC, GE, DA, Myoclonic, AA	Mean 68% reduction
	Cukiert et al. (80)	13	GE	≥50% reduction in 90% of patients
PM	Filipescu et al. (70)	8	T	Clinically “less severe” seizures with stimulation in 5/8
**Closed loop stimulation**				
ANT	Elder et al. (55)	3	Multifocal	≥50% reduction in 2/3 and 50% reduction in the third
	Herlopian et al. (54)	1	GE, myoclonic, DA, tonic, A	90–95% reduction at 2 years post-RNS
CM	Kokkinos et al. (64)	1	A, eyelid myoclonia	84% reduction
	Burdette et al. (65)	7	SP, CP, focal to bilateral tonic-clonic	Median 88% reduction
	Welch et al. (63)	1	A	75% reduction
PM	Burdette et al. (71)	3	SP, CP, focal to bilateral tonic-clonic	≥50% reduction in 3/3 and ≥90% reduction in 2/3

*ANT, Anterior Thalamic Nucleus; CM, Centromedian Nucleus; PM, Medial Pulvinar Nucleus; CP, Complex Partial Seizures; GTC, Generalized Tonic Clonic Seizures; DA, Drop Attacks; SGTC, Secondarily Generalized Tonic Clonic Seizures; AA, Atypical Absence Seizures; SP, Simple Partial Seizures; GE, generalized epilepsy; P, Partial Seizures; LGS, Lennox-Gastaut Syndrome; T, Temporal Epilepsy; A, Absence Seizures; F, Frontal Epilepsy; PA, Parietal Epilepsy; DBS, Deep Brain Stimulation; RNS, Responsive Neurostimulation*.

### Choosing Thalamic Stimulation Strategy

While open loop ANT stimulation is the most established target for epilepsy, recent studies have shown promise with deployment of closed loop stimulation (Table 1). Because patients with medically resistant epilepsy who are candidates for neuromodulation may be suitable for open or closed loop stimulation, selecting the optimal therapy can be challenging. The primary advantage of closed loop stimulation is the ability to provide a more personalized approach to care by configuring stimulation in response to the patient's specific needs and epileptic activity. However, this is limited by our current insufficient understanding of optimal stimulation parameters and electrode selection, and thus prevents maximal efficacy with a closed loop approach (84). In comparison to open loop stimulation, closed loop stimulation carries a lower burden of stimulation, fewer stimulation related side effects, fewer cognitive and mood disruptions, and records chronic ambulatory EEG data. It is also useful in measuring seizure burden in response to changes in antiepileptic medications, behavior modification, and in characterizing neurobehavioral spells. With utilization of open loop stimulation, the complexities of precise seizure localization and seizure detection algorithm set up required for closed loop stimulation can be avoided (85). A drawback of open loop stimulation, however, is the lack of capability to personalize therapy. As both open and closed loop stimulation have been shown to be efficacious and safe in managing medically refractory epilepsy, treatment decision making should be guided by patient specific factors.

## Conclusion

Over the past two decades, neuromodulatory techniques have demonstrated significant success in treating epilepsy in those who are refractory to medication or not suitable for traditional resective or ablative surgery. Current trends in the literature suggest that modulation of various thalamic nuclei, through open and closed loop systems, is an effective and safe option for these patients.

## Author Contributions

MA: ideation and manuscript writing. ND: manuscript writing. RA: supervision, editing, and manuscript writing. All authors contributed to the article and approved the submitted version.

## Conflict of Interest

The authors declare that the research was conducted in the absence of any commercial or financial relationships that could be construed as a potential conflict of interest.

## Publisher's Note

All claims expressed in this article are solely those of the authors and do not necessarily represent those of their affiliated organizations, or those of the publisher, the editors and the reviewers. Any product that may be evaluated in this article, or claim that may be made by its manufacturer, is not guaranteed or endorsed by the publisher.
